# Supplementary material for: Composition, Distribution and Mobility Potential of the Antibiotic Resistome in Sediments from the East China Sea Revealed by Metagenomic Analysis
Source: Microorganisms. 2025 Mar 20;13(3):697. doi: 10.3390/microorganisms13030697 (PMC11944410; doi:10.3390/microorganisms13030697)
Supplement: Supplementary file 1 [file microorganisms-13-00697-s001.zip › Supplementary data/Supplementary data.pdf]

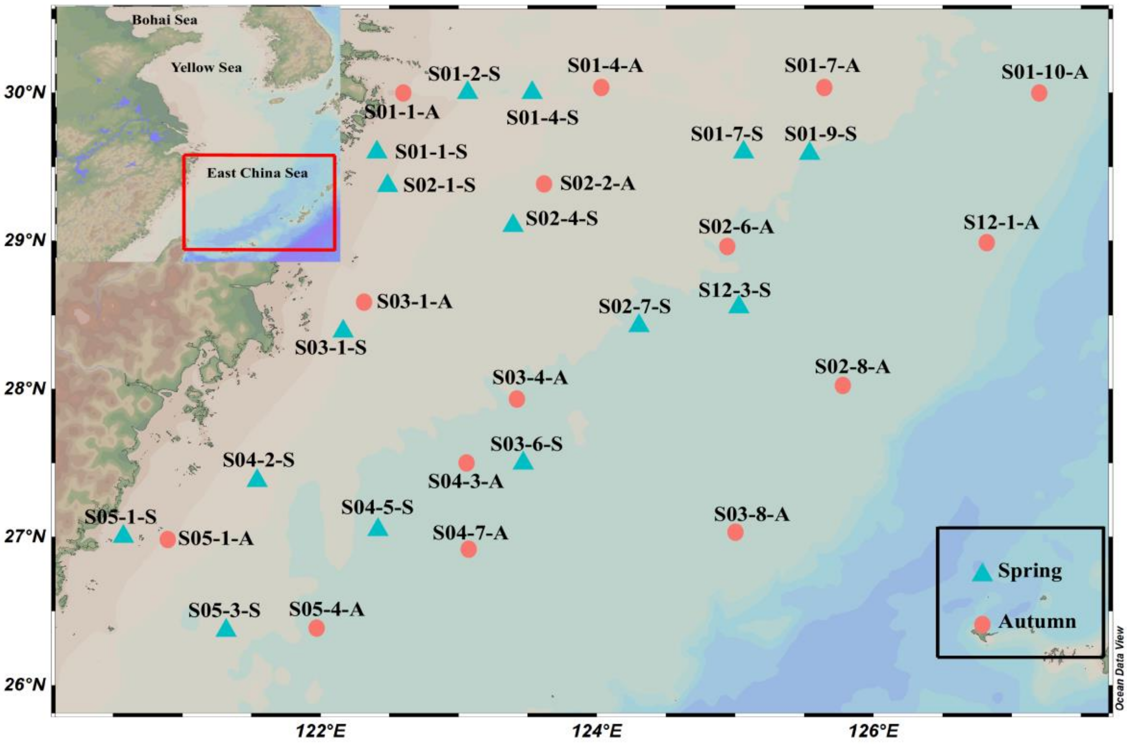

Figure S1. Sampling stations in the East China Sea.

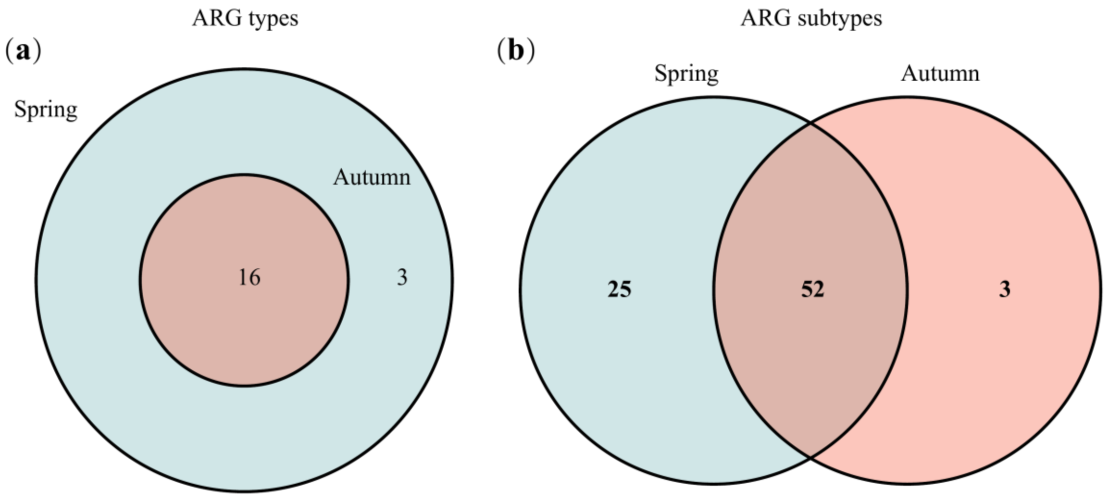

Figure S2. The Venn diagram of (a) ARG types and (b) subtypes across seasons.

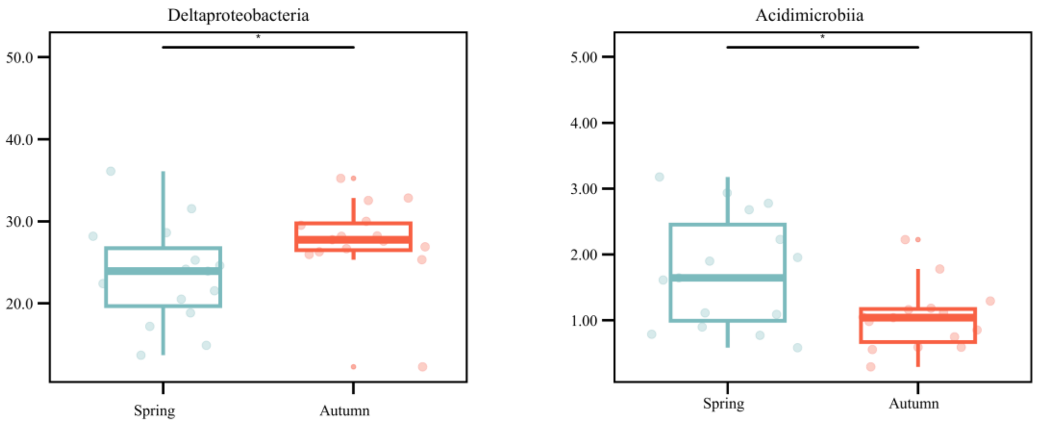

Figure S3. Boxplots showing the difference in abundance of ARG hosts between spring and autumn. The differences between the two groups were all compared by Student's *t* test. *** ≤ 0.001, ** ≤ 0.01, * ≤ 0.05, NS > 0.05.

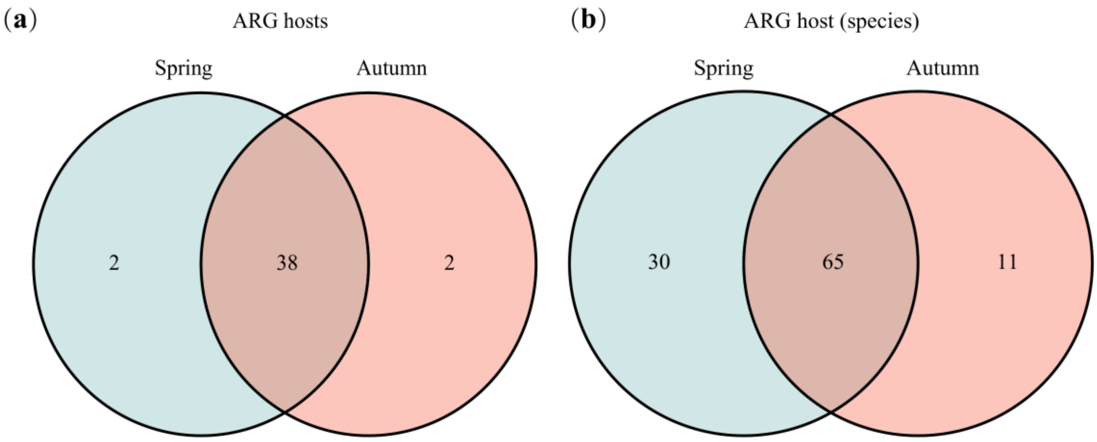

Figure S4. The Venn diagram of ARG hosts at the (a) class and (b) species level across seasons.

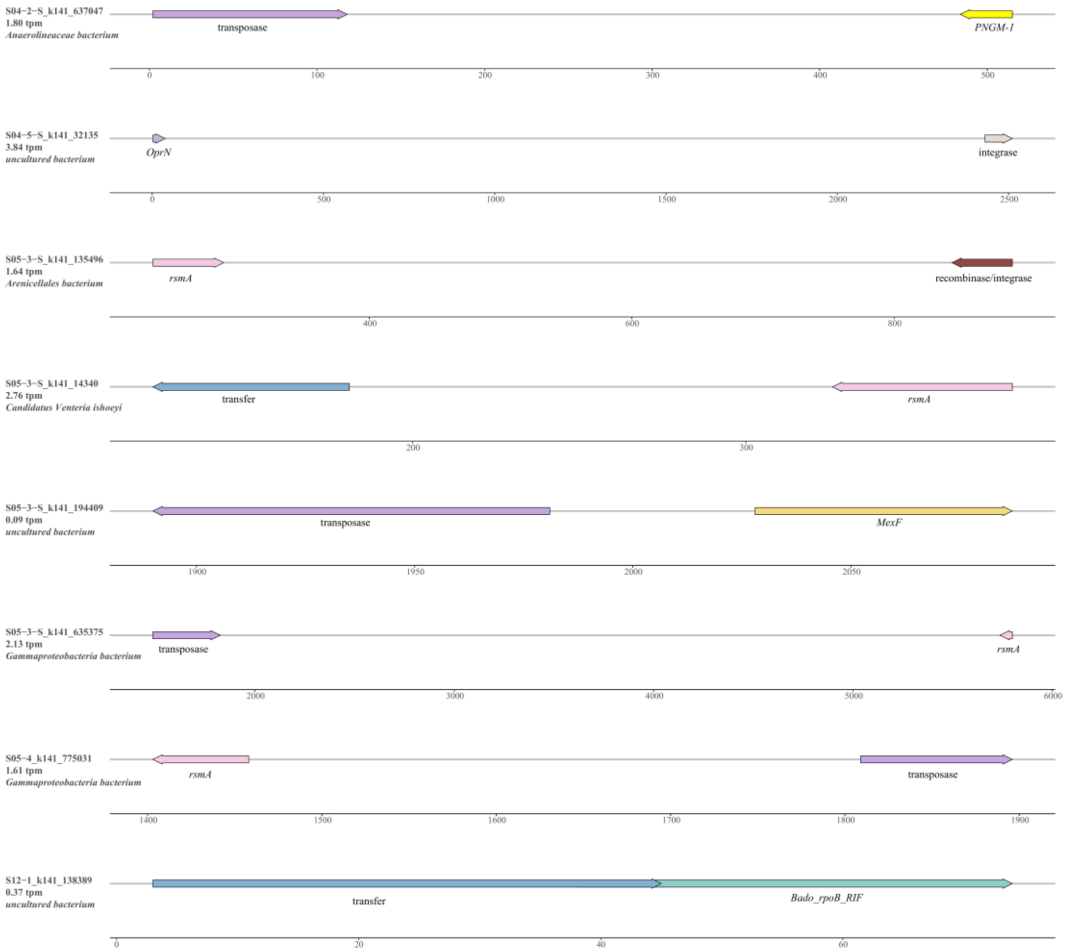

Figure S5. The co-existence arrangements among ARGs and MGEs at the contig level.

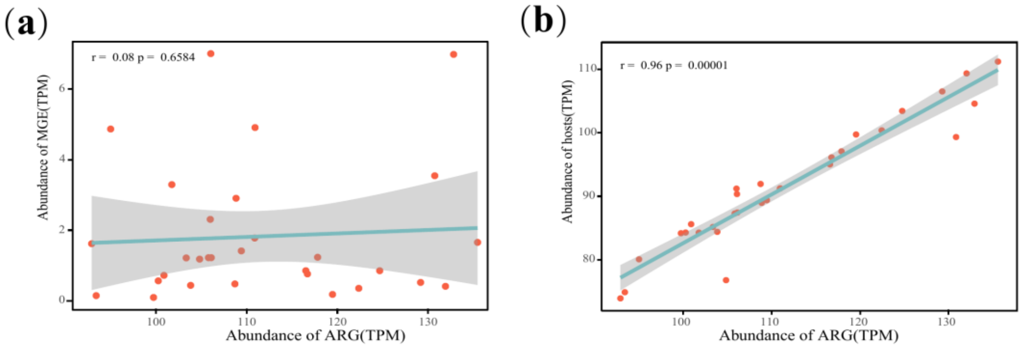

Figure S6. Correlation of (a) MGEs and (b) ARG hosts with the ARGs in the study region, correlations were computed using spearman, and shaded areas indicate 95% confidence intervals.

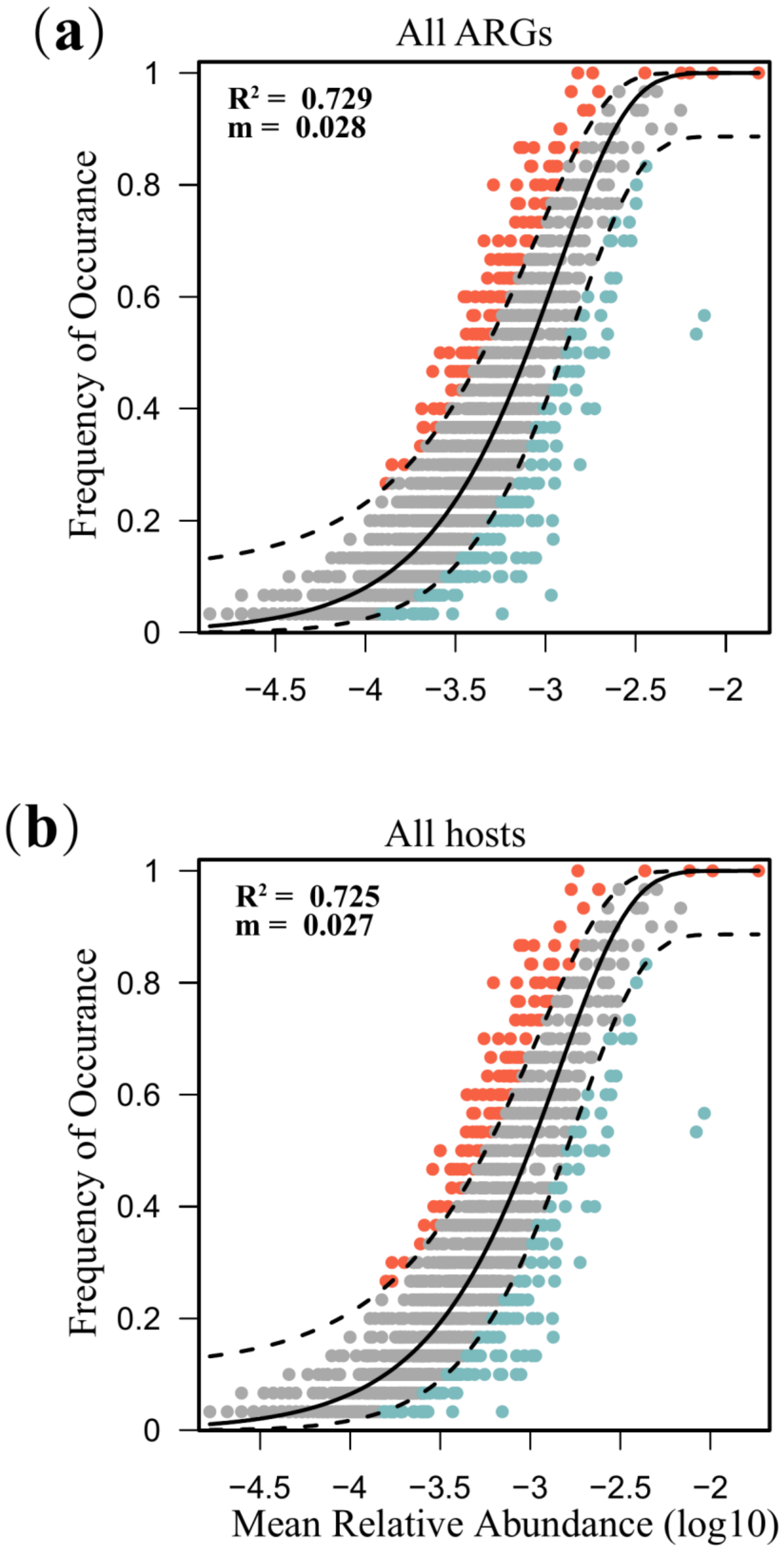

Figure S7. The NCM analysis of (a) ARGs and their (b) hosts.
